# Delayed fluorescence from inverted singlet and triplet excited states

**DOI:** 10.1038/s41586-022-05132-y

**Published:** 2022-09-14

**Authors:** Naoya Aizawa, Yong-Jin Pu, Yu Harabuchi, Atsuko Nihonyanagi, Ryotaro Ibuka, Hiroyuki Inuzuka, Barun Dhara, Yuki Koyama, Ken-ichi Nakayama, Satoshi Maeda, Fumito Araoka, Daigo Miyajima

**Affiliations:** 1 RIKEN Center for Emergent Matter Science (CEMS), Wako, Japan; 2 Department of Applied Chemistry, Graduate School of Engineering, Osaka University, Suita, Japan; 3 Precursory Research for Embryonic Science and Technology (PRESTO), Japan Science and Technology Agency (JST), Kawaguchi, Japan; 4 Graduate School of Organic Materials Science, Yamagata University, Yonezawa, Japan; 5 Department of Chemistry, Faculty of Science, Hokkaido University, Sapporo, Japan; 6 Institute for Chemical Reaction Design and Discovery (WPI-ICReDD), Hokkaido University, Sapporo, Japan

**Keywords:** Physical chemistry, Organic LEDs

## Abstract

Hund’s multiplicity rule states that a higher spin state has a lower energy for a given electronic configuration^1^. Rephrasing this rule for molecular excited states predicts a positive energy gap between spin-singlet and spin-triplet excited states, as has been consistent with numerous experimental observations over almost a century. Here we report a fluorescent molecule that disobeys Hund’s rule and has a negative singlet–triplet energy gap of −11 ± 2 meV. The energy inversion of the singlet and triplet excited states results in delayed fluorescence with short time constants of 0.2 μs, which anomalously decrease with decreasing temperature owing to the emissive singlet character of the lowest-energy excited state. Organic light-emitting diodes (OLEDs) using this molecule exhibited a fast transient electroluminescence decay with a peak external quantum efficiency of 17%, demonstrating its potential implications for optoelectronic devices, including displays, lighting and lasers.

## Abstract (web summary)

A fluorescent molecule is described that does not follow Hund’s rule and instead shows singlet and triplet excited states with inverted energy levels, leading to high-efficiency OLEDs with potential implications for optoelectronic devices.

## Main

The spin multiplicity of molecular excited states plays a crucial role in organic optoelectronic devices. In the case of OLEDs, recombination of charge carriers leads to the formation of singlet and triplet excited states in a 1:3 ratio. This spin statistics limits the internal quantum efficiency of OLEDs and leads to the energy loss owing to the spin-forbidden nature of triplet excited states to emit photons. To overcome this issue, two strategies for harvesting the ‘dark’ triplet excited states as photons have been established. The first relies on organometallic complexes with transition metals, such as iridium and platinum, which induce a large spin–orbit coupling to allow triplet states to emit photons as phosphorescence^2–4^. The other uses organic molecules that exhibit thermally activated delayed fluorescence (TADF)^5–7^. This class of materials has energetically close singlet and triplet excited states, in which ambient thermal energy upconverts the triplet states into the singlet states through reverse intersystem crossing (RISC). Although the concept of TADF has the advantage of eliminating the need for transition metals, the resultant temporally delayed fluorescence typically has a time constant in the microsecond or even millisecond range, which is long enough for detrimental bimolecular annihilations, such as triplet–triplet annihilation and triplet–polaron annihilation, to compete with delayed fluorescence. These bimolecular annihilations lead to the decrease in device efficiency under high current densities, known as efficiency roll-off in OLEDs^8,9^, and also generate high-energy excitons that are suspected to cause chemical degradation of materials, particularly in blue OLEDs^10^. The research community has thus focused on minimizing the singlet–triplet energy gap (Δ*E*_ST_) to accelerate the upconversion by thermal activation^7^. Alternatively, an ideal case would be thermodynamically favourable downconversion with negative Δ*E*_ST_, which is not expected if applying Hund’s multiplicity rule to the lowest-energy excited state. Herein, we demonstrate experimental evidence of the existence of highly fluorescent organic molecules that disobey Hund’s rule and possess negative Δ*E*_ST_ for constructing efficient OLEDs.

**Fig. 1:**
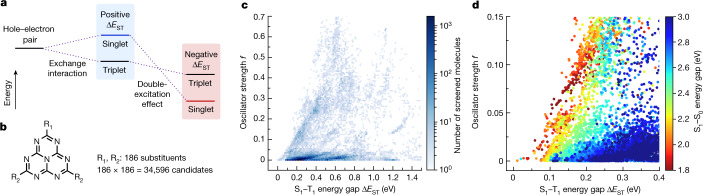
Computational screening of heptazine analogues. **a**, Schematic diagram of singlet and triplet excited states split in energy by the exchange interaction (middle) and then inverted by including the double-excitation effect (right). **b**, Molecular structures of the heptazine analogues examined in the computational screening. **c**, Number of screened molecules as a function of Δ*E*_ST_ and *f* calculated by TDDFT. **d**, S_1_–S_0_ energy gaps as a function of Δ*E*_ST_ and *f* calculated by TDDFT.

Numerous observations of positive Δ*E*_ST_ in molecular excited states are generally understood by the exchange interaction, the quantum-mechanical effect involving Pauli repulsion, which stabilizes triplet states relative to singlet states^11^. Δ*E*_ST_ is simply equal to twice the positive exchange energy if the lowest-energy singlet and triplet excited states (S_1_ and T_1_) have the same single-excitation configuration^11^. Although there is general agreement that Δ*E*_ST_ must be positive, potentially negative Δ*E*_ST_ has been discussed in nitrogen-substituted phenalene analogues, such as cycl[3.3.3]azine and heptazine, during the past two decades^12–21^. Recent theoretical studies have also suggested the possibility of negative Δ*E*_ST_ in these molecules by accounting for double-excitation configurations in which two electrons of occupied orbitals have been promoted out to virtual orbitals^15–19^ (Supplementary Fig. 1). Because the Pauli exclusion principle restricts the accessible double-excitation configurations in T_1_, an effective admixture of such configurations stabilizes S_1_ relative to T_1_. If this stabilization overcomes the exchange energy, Δ*E*_ST_ could be a negative value (Fig. 1a). However, to the best of our knowledge, none of the molecules has been experimentally identified with negative Δ*E*_ST_ and the resultant delayed fluorescence from inverted singlet and triplet excited states (DFIST). We note that the accounting for double-excitation configurations has proved crucial to theoretically reproduce the small but positive Δ*E*_ST_ of 5,9-diphenyl-5,9-diaza-13b-boranaphtho[3,2,1-*de*]anthracene (DABNA-1) (0.15 eV)^22,23^.

Pioneering computational calculations^15^ inspired us to focus on heptazine as a potential class of molecules that exhibit DFIST. Correlated wave function theories suggested that S_1_ of heptazine lies 0.2–0.3 eV below T_1_, although S_1_ is a ‘dark’ state, meaning that the electronic transition to the ground state (S_0_) is dipole-forbidden and the oscillator strength (*f*) is zero in the *D*_3h_ symmetry point group. Notably, the heptazine core is shared by several synthesized molecules that exhibit intense TADF^24,25^ with positive Δ*E*_ST_ (refs. ^26,27^). Furthermore, the recent computational screening by Pollice et al. has demonstrated that appropriate chemical modifications of heptazine recover *f* while retaining negative Δ*E*_ST_ (ref. ^19^). As such, we introduced 186 different substituents to heptazine to generate 34,596 candidate molecules for computational screening. The structures of all substituents are available in Supplementary Fig. 2. To ensure the synthetic feasibility, at most two distinct types of substituents were introduced to the heptazine core as R_1_ and R_2_ (Fig. 1b). We used standard linear-response time-dependent density functional theory (TDDFT) to calculate Δ*E*_ST_ and *f*, which are more affordable in computational cost than those calculated by correlated wave function theories. Although the commonly used adiabatic approximation in TDDFT does not account for double-excitation character^16,28^, the properties calculated by TDDFT are still useful to prescreening for narrowing the list of the candidate molecules before the high-cost calculations and experimental evaluation, as both S_1_ and T_1_ of heptazine are almost dominated by the single-excitation configuration between the highest occupied molecular orbital (HOMO) and the lowest unoccupied molecular orbital (LUMO)^15^.

**Fig. 2:**
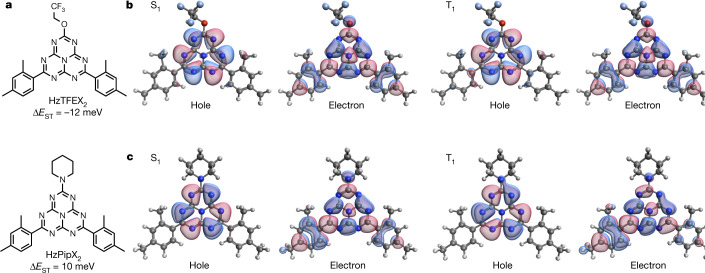
Lead candidate molecules HzTFEX_2_ and HzPipX_2_. **a**, Molecular structures of HzTFEX_2_ and HzPipX_2_ with Δ*E*_ST_ calculated by EOM-CCSD. **b**,**c**, Dominant pair of NTOs of S_1_ and T_1_ of HzTFEX_2_ (**b**) and HzPipX_2_ (**c**) for the EOM-CCSD calculations.

Figure 1c shows the statistics of the screened molecules as a function of Δ*E*_ST_ and *f* calculated by TDDFT. A well-known trade-off between small Δ*E*_ST_ and large *f* is evident from this particular dataset of heptazine analogues. Although balancing such a trade-off is a key concern in recent synthetic efforts on TADF materials, Fig. 1c demonstrates the optimal combinations of Δ*E*_ST_ and *f* for which one parameter can no longer be improved without sacrificing the other. Figure 1d further visualizes the trade-off between Δ*E*_ST_ and *f* for each fluorescence colour. The screening data suggest 5,264 promising candidates to show fluorescence across the entire visible spectrum, with Δ*E*_ST_ < 0.35 eV and *f* > 0.01. Setting the range of the vertical S_1_–S_0_ energy gap to 2.70–2.85 eV for blue fluorescence further narrows down the candidates to 1,028 molecules, corresponding to 2.97% of all the screened molecules. We then assessed their synthetic feasibility and selected two heptazine analogues HzTFEX_2_ and HzPipX_2_ (Fig. 2a) for further evaluation. We note that these molecules recover *f* while retaining small Δ*E*_ST_ (*f* = 0.010 and 0.015 and Δ*E*_ST_ = 210 and 334 meV for HzTFEX_2_ and HzPipX_2_, respectively). This trend is consistent with the recent computational screening on heptazine analogues with asymmetrical substitutions^19^.

To examine whether HzTFEX_2_ and HzPipX_2_ could have negative Δ*E*_ST_, we computed their S_1_ and T_1_ by correlated wave function theories. Equation-of-motion coupled cluster with single and double excitation (EOM-CCSD)^29^ calculations predict HzTFEX_2_ to possess negative Δ*E*_ST_ of −12 meV, affirming its potential for exhibiting DFIST. In comparison, Δ*E*_ST_ calculated for HzPipX_2_ remains at a positive value of 10 meV, which is comparable with those of the current state-of-the-art TADF materials^30–38^. Figure 2b,c shows the dominant pair of natural transition orbitals (NTOs)^39^ for S_1_ and T_1_. In both molecules, the hole orbitals are exclusively localized on the peripherical six nitrogen atoms of the heptazine core, whereas the electron orbitals are localized on the central nitrogen atom and the carbon atoms of the core, as well as on the substituents. The spatial separation of these orbitals indicates that the exchange interaction is weak, resulting in nearly degenerate S_1_ and T_1_ in the single-excitation picture. Similar spatial separations of NTOs have also been found in the multi-resonant TADF materials, such as DABNA-1 (refs. ^22,23^). In this situation, the stabilization of S_1_ by including the double-excitation configurations becomes more dominant to determine the sign of Δ*E*_ST_. Indeed, S_1_ of both molecules comprise double-excitation configurations with weights of around 1% described as the sum of the squares of the doubles amplitudes in EOM-CCSD, which are slightly higher than those of T_1_. Two other wave-function-based calculations using second-order algebraic diagrammatic construction (ADC(2))^40^ and complete active space with second-order perturbation theory (CASPT2)^41^ further validate the inversion of S_1_ and T_1_ in HzTFEX_2_ with calculated Δ*E*_ST_ of −34 meV and −184 meV, respectively. However, for HzPipX_2_, the two methods also invert Δ*E*_ST_ (−12 meV with ADC(2) and −171 meV with CASPT2) as compared with the positive value of 10 meV predicted with EOM-CCSD (Supplementary Table 1). This variation in estimates of Δ*E*_ST_ highlights the current limitations of excited-state calculations and demands conclusive experimental evaluation. We note that Δ*E*_ST_ calculated by other second-order methods are given in the Supplementary Information, as well as the dependence of the choice of the guess orbitals and the size of the active space on the CASPT2 results.

**Fig. 3:**
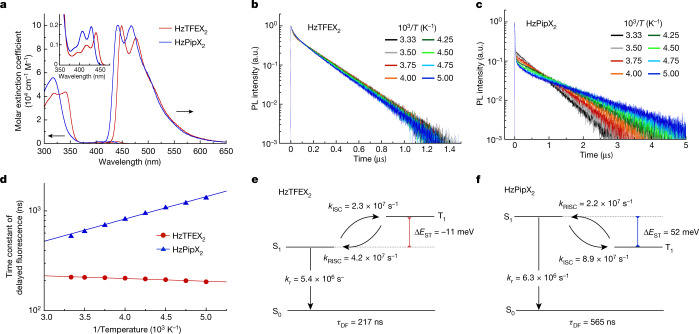
Photophysical properties of HzTFEX_2_ and HzPipX_2_ in deaerated toluene solutions. **a**, Steady-state absorption and PL spectra of HzTFEX_2_ and HzPipX_2_. The inset is the magnified view of the absorption spectra. **b**,**c**, Transient PL decays of HzTFEX_2_ (**b**) and HzPipX_2_ (**c**) at varying temperatures. **d**, Temperature dependence of *τ*_DF_ of HzTFEX_2_ and HzPipX_2_; the solid lines in **d** represent the fits of *τ*_DF_ to a single exponential in inverse temperature. **e**,**f**, Schematic diagram of the excited states and the associated transitions of HzTFEX_2_ (**e**) and HzPipX_2_ (**f**).

**Extended Data Fig. 1:**
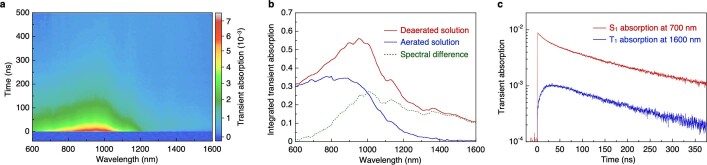
Transient absorption data of HzTFEX_2_. **a**, Transient absorption of HzTFEX_2_ as a function of wavelength and time in a deaerated toluene solution. **b**, Integrated transient absorption spectra of HzTFEX_2_ over 0–500 ns in deaerated and aerated toluene solutions; the dashed green dashed line represents their spectral difference and mainly corresponds to the transient absorption of T_1_. **c**, Transient absorption decays of S_1_ and T_1_ monitored at 700 nm and 1,600 nm, respectively.

HzTFEX_2_ and HzPipX_2_ were synthesized by nucleophilic aromatic substitution of 2,5,8-trichloroheptazine with corresponding alcohol or amine, followed by Friedel–Crafts reactions with *m*-xylene. The details of the synthesis and characterization are given in the Supplementary Information. The photophysical properties of the two molecules were evaluated in deaerated toluene solutions (Fig. 3a and Extended Data Table 1). The steady-state absorption spectra of HzTFEX_2_ and HzPipX_2_ comprise the lowest-energy absorption band centred at 441 nm and 429 nm, respectively, with small molar absorption coefficients on the order of 10^3^ M^−1^ cm^−1^, reflecting the spatial separation between the hole and electron NTOs computed for S_1_ of each molecule. On photoexcitation, HzTFEX_2_ exhibits blue emission with a peak wavelength (*λ*_PL_) of 449 nm and a photoluminescence (PL) quantum yield (*Φ*_PL_) of 74%, whereas slightly blue-shifted *λ*_PL_ of 442 nm and similar *Φ*_PL_ of 67% are observed for HzPipX_2_. These energy differences in absorption and emission are also predicted by TDDFT calculations and are attributed to the stronger electron-donating effect of the piperidyl group in HzPipX_2_ than that of 2,2,2-trifluoroethoxy group in HzTFEX_2_. In aerated toluene solutions, *Φ*_PL_ of HzTFEX_2_ and HzPipX_2_ decrease to 54% and 37%, respectively. Because atmospheric O_2_ can quench molecular triplet excited states and the change in *Φ*_PL_ is reversible, we ascribe the blue emissions of the two molecules, at least partially, to delayed fluorescence through forward intersystem crossing (ISC) and RISC between S_1_ and T_1_. This assumption is supported by transient absorption decay measurements on HzTFEX_2_, which scrutinized ISC from S_1_ to T_1_ as the signal decay of S_1_ at 700 nm and the signal growth of T_1_ at 1,600 nm, followed by the persistent signal decays of both S_1_ and T_1_ (Extended Data Fig. 1). We also note that both decays have similar time constants (223 ns for S_1_ and 210 ns for T_1_), indicating the steady-state condition with the constant population ratio maintained by ISC and RISC.

To show the excited-state kinetics of the two molecules in detail, we performed transient PL decay measurements at varying temperatures (Fig. 3b,c and Supplementary Fig. 3 for the log–log representation). Both molecules exhibit biexponential transient PL decays, which comprise nanosecond-order prompt fluorescence followed by sub-microsecond delayed fluorescence with temperature-dependent time constants. Remarkably, the time constant of delayed fluorescence (*τ*_DF_) of HzTFEX_2_ gradually decreases from 217 ns to 195 ns with decreasing temperature from 300 K to 200 K (Fig. 3d). This anomalous temperature dependence of *τ*_DF_ indicates that S_1_ lies energetically below T_1_, for which lowering the temperature shifts the steady-state population towards emissive S_1_ relative to dark T_1_ and thus accelerates the delayed fluorescence (that is, decreases *τ*_DF_). In comparison, *τ*_DF_ of HzPipX_2_ increases from 565 ns to 1,372 ns by the same temperature decrease, as has been similarly observed in conventional TADF materials^5–7^. It is worth noting that *τ*_DF_ of HzTFEX_2_ is much shorter than emission time constants ever reported for TADF materials^30–38^ and phosphorescent materials^2–4^ used for efficient OLEDs, which are typically in the microsecond range.

**Extended Data Fig. 2:**
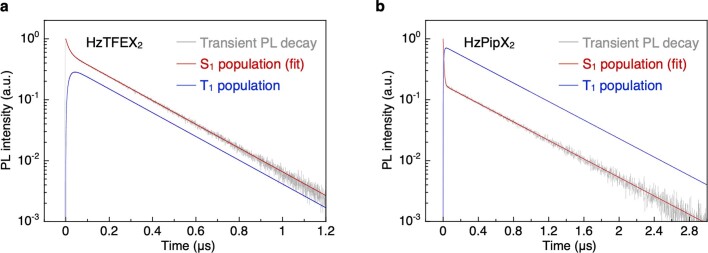
Analysis of the transient PL decays of HzTFEX_2_ and HzPipX_2_. **a**,**b**, Fit (solid red line) of the S_1_ population in equation (1) to the transient PL decay of HzTFEX_2_ (**a**) and HzPipX_2_ (**b**) in deaerated toluene solutions at 300 K. The solid blue line represents the T_1_ population simulated by the best-fit parameters *k*_r_ + *k*_nr_, *k*_ISC_ and *k*_RISC_ in equation (1).

**Extended Data Fig. 3:**
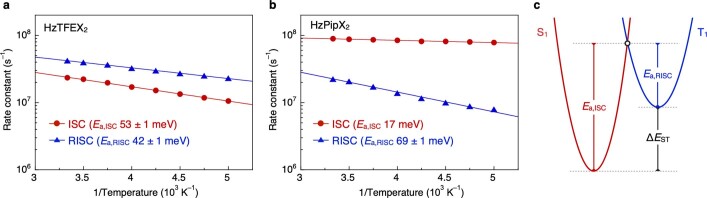
*k*_ISC_ and *k*_RISC_ of HzTFEX_2_ and HzPipX_2_. **a**,**b**, Temperature dependence of *k*_ISC_ and *k*_RISC_ of HzTFEX_2_ (**a**) and HzPipX_2_ (**b**) in deaerated toluene. The solid lines in **a** and **b** represent the fits of the plots to the Arrhenius equation. The error bars of the plots in **a** and **b** are smaller than the plot size. **c**, Schematic diagram of the potential energy surfaces of S_1_ and T_1_ and the activation energies of ISC and RISC.

**Extended Data Fig. 4:**
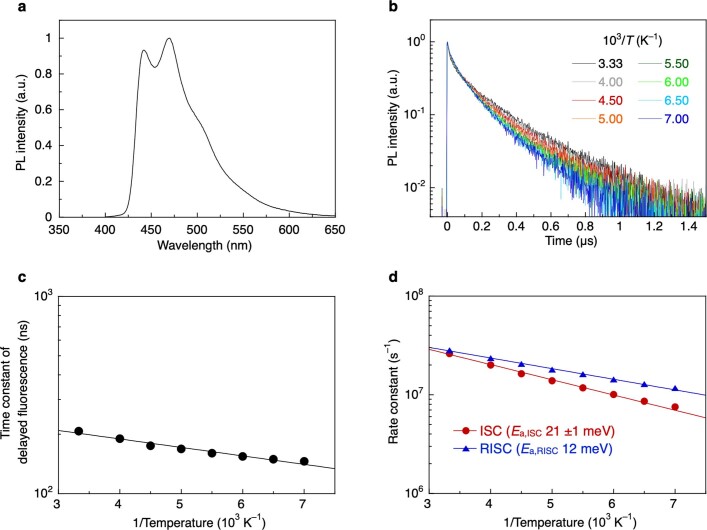
Photophysical properties of HzTFEX_2_ in a solid-state host matrix. **a**, Steady-state PL spectra of a thin film of bis(diphenylphosphoryl)dibenzo[*b*,*d*]furan (PPF):1 wt% HzTFEX_2_. **b**, Transient PL decays of PPF:1 wt% HzTFEX_2_ at varying temperatures under a N_2_ atmosphere. **c**, Temperature dependence of *τ*_DF_ of PPF:1 wt% HzTFEX_2_. **d**, Temperature dependence of *k*_ISC_ and *k*_RISC_ of PPF:1 wt% HzTFEX_2_. The solid lines in **d** represent the fits of the plots to the Arrhenius equation.

We further analysed the temperature-dependent PL decay kinetics with the underlying rate equation. In the absence of phosphorescence and non-radiative decay of T_1_ to S_0_, the rate equation for the populations of S_1_ and T_1_ is given by

1 $$\frac{{\rm{d}}}{{\rm{d}}t}\left(\begin{array}{c}{S}_{1}\\ {T}_{1}\end{array}\right)=\left(\begin{array}{cc}-({k}_{{\rm{r}}}+{k}_{{\rm{nr}}}+{k}_{{\rm{ISC}}}) & {k}_{{\rm{RISC}}}\\ {k}_{{\rm{ISC}}} & -{k}_{{\rm{RISC}}}\end{array}\right)\left(\begin{array}{c}{S}_{1}\\ {T}_{1}\end{array}\right)$$

in which *k*_r_, *k*_nr_, *k*_ISC_ and *k*_RISC_ are the rate constants of radiative decay of S_1_ to S_0_, non-radiative decay of S_1_ to S_0_, ISC of S_1_ to T_1_ and RISC of T_1_ to S_1_, respectively. By numerically fitting equation (1) to the PL decay data at 300 K, we found that RISC is faster than ISC in HzTFEX_2_ (*k*_RISC_ = 4.2 × 10^7^ s^−1^ versus *k*_ISC_ = 2.3 × 10^7^ s^−1^), whereas RISC is slower than ISC in HzPipX_2_ (*k*_RISC_ = 2.2 × 10^7^ s^−1^ versus *k*_ISC_ = 8.9 × 10^7^ s^−1^) (Fig. 3e,f). These parameters simulate that the population of T_1_ is lower than that of S_1_ in HzTFEX_2_ under the steady-state condition, indicating that S_1_ lies energetically below T_1_ (Extended Data Fig. 2). Furthermore, the temperature dependence of *k*_ISC_ and *k*_RISC_ follows the Arrhenius equation, *k* = *A*exp(−*E*_a_/*k*_B_*T*), in which *k* is the rate constant, *A* is the pre-exponential factor, *E*_a_ is the activation energy, *k*_B_ is the Boltzmann constant and *T* is the absolute temperature (Extended Data Fig. 3). The best-fit parameters of the Arrhenius equation yield the activation energies of ISC and RISC (*E*_a,ISC_ and *E*_a,RISC_) (Extended Data Table 1). Subtracting *E*_a,ISC_ from *E*_a,RISC_, we determined Δ*E*_ST_ of HzTFEX_2_ to be −11 ± 2 meV, which is in marked contrast to positive Δ*E*_ST_ ever observed in numerous molecules, as well as in HzPipX_2_ (Δ*E*_ST_ = 52 ±1 meV). We note that the change in *k*_r_ + *k*_nr_ at varying temperatures is negligible compared with those in *k*_ISC_ and *k*_RISC_ (Supplementary Fig. 4) and thus the decreasing trend of *τ*_DF_ of HzTFEX_2_ is more reasonably attributed to the inverted S_1_ and T_1_. The negative Δ*E*_ST_ of HzTFEX_2_ is retained in a solid-state host matrix (see Extended Data Fig. 4 and the Supplementary Information for details).

Having experimentally determined negative Δ*E*_ST_, we conclude that HzTFEX_2_ exhibits DFIST. Further synthetic efforts replacing the xylyl groups in HzTFEX_2_ with either phenyl or tolyl groups led to HzTFEP_2_ and HzTFET_2_, which similarly show DFIST with measured Δ*E*_ST_ of −14 ± 3 meV and −13 ± 3 meV, respectively (see Extended Data Table 1 and Supplementary Fig. 5 for details), indicating the potential of heptazines for further developing efficient DFIST materials. In common with the three materials, ISC from S_1_ to T_1_ competes with the inherently slow radiative decay of heptazines, followed by faster RISC, leading to a significant S_1_ population relative to T_1_ and sub-microsecond DFIST. Thus, we propose to refer to the present type of emissions as ‘H (heptazine)-type delayed fluorescence’ by analogy with ‘E (eosin)-type delayed fluorescence’ referred to as TADF^42^ and ‘P (pyren)-type delayed fluorescence’ involving triplet–triplet annihilation^43^.

**Fig. 4:**
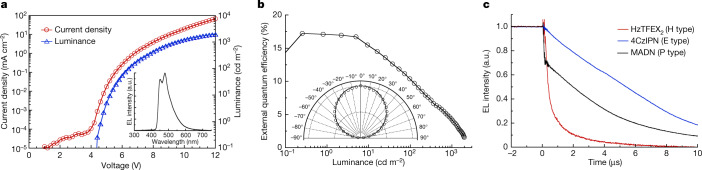
OLED performance. **a**,**b**, Current density–voltage–luminance characteristics (**a**) and external quantum efficiency–luminance characteristics (**b**) of the fabricated OLED using HzTFEX_2_; the inset in **a** shows the EL spectra measured at 1.0 mA and the inset in **b** represents the viewing-angle dependence of the luminance, which is almost consistent with the Lambertian distribution. **c**, Transient EL decays of the OLEDs using HzTFEX_2_, 4CzIPN and MADN, respectively, measured in pulse operation with square-wave voltages of 8 V and −4 V.

Finally, we evaluated the electroluminescence (EL) properties of HzTFEX_2_ in OLEDs fabricated by thermal evaporation. The details of the fabrication procedures and the device structures are given in the Supplementary Information. Figure 4a,b shows the EL spectra, current density–voltage–luminance characteristics and external quantum efficiency–luminance characteristics of the OLED. Intense blue EL originating from HzTFEX_2_ was observed with spectral peak wavelengths (*λ*_EL_) at 450 nm and 479 nm and Commission internationale de l’éclairage (CIE) coordinates of (0.17, 0.24). The maximum external quantum efficiency reached 17%, corresponding to the internal quantum efficiency of 80% for a bottom-emission OLED with a typical light-outcoupling efficiency of 20% ^44^. We note that the viewing-angle dependence of the luminance followed the Lambertian distribution (Fig. 4b inset), ensuring accurate estimation of the external quantum efficiency from the forward emission. Remarkably, HzTFEX_2_ exhibited fast transient EL decay, reflecting the sub-microsecond H-type delayed fluorescence (Fig. 4c). In comparison, much slower transient EL decays were observed for E-type delayed fluorescence of 2,4,5,6-tetra(carbazol-9-yl)isophthalonitrile (4CzIPN)^6^ and P-type delayed fluorescence of 2-methyl-9,10-bis(naphthalen-2-yl)anthracene (MADN)^45^, although the EL of MADN initially decayed faster by the prompt fluorescence solely from S_1_ (ref. ^46^). It is thus evident that the fast triplet harvesting of HzTFEX_2_ with negative Δ*E*_ST_ can be retained even in actual OLEDs. Although the efficiency roll-off is still marked in this preliminary device concerning the large hole injection barrier caused by the high ionization potential of HzTFEX_2_ (6.3 eV), we anticipate that further optimization of molecular design will address this issue and allow a conclusive exploration of the effects of negative Δ*E*_ST_ on efficiency roll-off and device stability.

In conclusion, we have demonstrated fluorescent heptazine molecules that possess negative Δ*E*_ST_. We observed their blue delayed fluorescence in both PL and EL with anomalous features: (1) the very short decay time constants (*τ*_DF_ ≈ 0.2 μs), (2) the decreasing trend of *τ*_DF_ with decreasing temperature and (3) the rate inversion of RISC and ISC (*k*_RISC_ > *k*_ISC_). These features indeed arise from negative Δ*E*_ST_ and led to the terminology ‘delayed fluorescence from inverted singlet and triplet excited states (DFIST)’ or ‘H (heptazine)-type delayed fluorescence’. We predict that further development of DFIST materials will offer stable and efficient OLEDs based on the fast triplet-to-singlet downconversion, with great implications for displays, lighting and lasers.

## Methods

### Quantum-chemical calculations

For the 34,596 heptazine molecules, the T_1_ geometries were optimized using spin-unrestricted DFT with the LC-BLYP functional and the 6-31G basis set. Vibrational frequency analysis for HzTFEX_2_, HzPipX_2_, HzTFEP_2_ and HzTFET_2_ gave no imaginary frequencies at the same level of theory. The vertical excitation energies of S_1_ and T_1_ were calculated using liner-response TDDFT with the LC-BLYP functional and the 6-31G(d) basis set within the Tamm–Dancoff approximation. The range-separation parameter of the LC-BLYP functional was non-empirically optimized to 0.18 bohr^−1^ to minimize the difference between the energy of the HOMO and the ionization potential of the neutral system and the difference between the energy of the HOMO of the radical anion system and the electron affinity of the neutral system^47^ of 2,5,8-triphenylheptazine. The T_1_ geometries of HzTFEX_2_ and HzPipX_2_ were also optimized using spin-unrestricted second-order Møller–Plesset perturbation theory (MP2) with the correlation consistent cc-pVDZ basis set. At the MP2 geometries of HzTFEX_2_ and HzPipX_2_, the vertical excitation energies of S_1_ and T_1_ were calculated using EOM-CCSD^29^, ADC(2)^40^ and CASPT2^41^ with the cc-pVDZ basis set. The CASPT2 calculations were performed with the fully internally contracted scheme over the state-averaged complete active space self-consistent field (CASSCF) wavefunctions with the active space of 12 electrons and 12 orbitals using the resolution of identity approximation with the auxiliary fitting basis set. The DFT, TDDFT, MP2 and EOM-CCSD calculations were performed using the Gaussian 16 Rev C.01 program. The ADC(2) calculations were performed using the Q-Chem 5.3.0 program. The CASSCF and CASPT2 calculations were performed using the Orca 4.2.1 program.

### Materials and synthesis

Commercially available reagents and solvents were used without further purification unless otherwise noted. 4CzIPN and MADN were purchased from Luminescence Technology Corporation and e-Ray Optoelectronics Technology, respectively. The synthetic procedures and characterization data of the heptazine molecules are detailed in the Supplementary Information.

### Photophysical measurements

Steady-state ultraviolet–visible absorption spectra were recorded on a Shimadzu UV-3600i Plus spectrophotometer. Steady-state PL spectra were acquired on a HORIBA FL3 spectrofluorometer with 370-nm photoexcitation from a Xe arc lamp. The absolute PL quantum yields were determined using a Hamamatsu Photonics C9920 integrated sphere system with 370-nm excitation from a Xe arc lamp. Transient absorption decay measurements were performed by a randomly interleaved plus train method^48^ on a UNISOKU picoTAS system with a 355-nm Q-switched laser pump source (pulse width <350 ps) and a supercontinuum white probe source (pulse width <100 ps). Transient PL decay measurements were performed by time-correlated single-photon counting on a HORIBA FL3 spectrofluorometer with a 370-nm LED pump source (pulse width <1.2 ns) and a UNISOKU CoolSpek cryostat using liquid nitrogen as the coolant. Ionization potentials were determined using a RIKEN KEIKI AC-3 ultraviolet photoelectron yield spectrometer.

### Analysis of transient PL decay kinetics

The time constants of prompt and delayed fluorescence (*τ*_PF_ and *τ*_DF_) were determined by biexponential decay fitting and deconvolution with the instrument response function. It is common when determining the rate constants of the transitions involved in TADF to assume *k*_ISC_ >> *k*_RISC_ such that the contribution of RISC to the prompt fluorescence is negligible^49^. However, this assumption does not hold true for DFIST materials with *k*_RISC_ > *k*_ISC_. Thus, *k*_r_ + *k*_nr_, *k*_ISC_ and *k*_RISC_ were determined without assuming *k*_ISC_ >> *k*_RISC_ by fitting the S_1_ population in equation (1) to the transient PL decay data using the scipy.integrate.odeint and scipy.optimize.curve_fit functions in Python 3.7^50^. *k*_r_ and *k*_nr_ were determined from *Φ*_PL_ = *k*_r_/(*k*_r_ + *k*_nr_) assuming negligible non-radiative decay of T_1_ to S_0_. Activation energies of ISC and RISC (*E*_a,ISC_ and *E*_a,RISC_) were determined by fitting the Arrhenius equation to the temperature dependence of *k*_ISC_ and *k*_RISC_, respectively. Δ*E*_ST_ was determined by subtracting *E*_a,ISC_ from *E*_a,RISC_.

### OLED fabrication and evaluation

The fabrication procedures of OLEDs are detailed in the Supplementary Information. EL spectra were recorded using a Hamamatsu Photonics PMA-12 photonic multichannel analyser. Current density–voltage–luminance characteristics were measured using a Konica Minolta CS-200 luminance meter and a Keithley 2400 source meter. The viewing-angle dependence of luminance was measured using a home-build spectro-goniometer with a Konica Minolta CS-2000 spectroradiometer. Transient EL decays measurements were performed using a home-build set-up with a Hamamatsu Photonics H7826 silicon photomultiplier tube (time response = 1.5 ns) and an Agilent 33220A function generator for pulse OLED operation (square-wave voltages = 8, −4 V and frequency = 2 kHz).

### Reporting summary

Further information on research design is available in the Nature Research Reporting Summary linked to this article.

## Online content

Any methods, additional references, Nature Research reporting summaries, source data, extended data, supplementary information, acknowledgements, peer review information; details of author contributions and competing interests; and statements of data and code availability are available at 10.1038/s41586-022-05132-y.

### Supplementary information

Supplementary Information This file contains Supplementary Figs. 1–12, Supplementary Materials and Methods and Supplementary Tables 1–12: see contents page for details.

Reporting Summary

Peer Review File

## Figures and Tables

**Extended Data Table 1:**
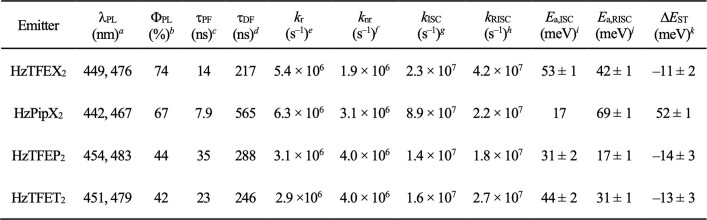
Photophysical properties of HzTFEX_2_ and HzPipX_2_ in deaerated toluene solutions

^a^Photoluminescence (PL) peak wavelength. ^b^PL quantum yield. ^c^Time constant of prompt fluorescence. ^d^Time constant of delayed fluorescence. ^e^Rate constant of radiative decay of the lowest-energy excited state (S_1_) to the ground state (S_0_). ^f^Rate constant of non-radiative decay of S_1_ to S_0_. ^g^Rate constant of intersystem crossing (ISC) of S_1_ to the lowest-energy triplet excited state (T_1_). ^h^Rate constant of reverse intersystem crossing (RISC) of T_1_ to S_1_. ^i^Activation energy of ISC. ^j^Activation energy of RISC. ^k^Energy gap between S_1_ and T_1_.

## Data Availability

The data underlying this article are available at 10.6084/m9.figshare.20058977.
