# Supplementary material for: Delayed fluorescence from inverted singlet and triplet excited states
Source: Nature. 2022 Sep 14;609(7927):502–6. doi: 10.1038/s41586-022-05132-y (PMC9477729; doi:10.1038/s41586-022-05132-y)
Supplement: Supplementary file 1 — This file contains Supplementary Figs. 1–12, Supplementary Materials and Methods and Supplementary Tables 1–12: see contents page for details. [file 41586_2022_5132_MOESM1_ESM.docx]

**Supplementary Information**

**Delayed Fluorescence from Inverted Singlet and Triplet Excited States**

Naoya Aizawa*, Yong-Jin Pu*, Yu Harabuchi, Atsuko Nihonyanagi, Ryotaro Ibuka, Hiroyuki Inuzuka_,_ Barun Dhara, Yuki Koyama, Ken-ichi Nakayama, Satoshi Maeda, Fumito Araoka and Daigo Miyajima*

Corresponding Authors: E-mail: aizawa@chem.eng.osaka-u.ac.jp; yongjin.pu@riken.jp; daigo.miyajima@riken.jp

**Table of contents**

**1. Supplementary synthesis and characterisation**

**2. Supplementary materials and methods**

**Supplementary Fig. 1** Electronic configurations for two electrons in three orbitals

**Supplementary Fig. 2** Structures of 186 substituents used for generating the candidate heptazine analogues

**Supplementary Table 1** Vertical S_1_ and T_1_ excitation energies, singlet–triplet energy gap (Δ*E*_ST_), and oscillator strength (*f*) of HzTFEX_2_ and HzPipX_2_, calculated by various methods

**Supplementary Fig. 3** Log-log representation of the transient PL decays of HzTFEX_2_ and HzPipX_2_ at varying temperatures

**Supplementary Fig. 4** Rate constants of radiative decay and non-radiative decay (*k*_r_ and *k*_nr_)

**Supplementary Fig. 5** Photophysical properties of HzTFEP_2_ and HzPipT_2_

**Supplementary Fig. 6** OLED performance

**Supplementary Table 2** Summary of the OLED performance

**3. Supplementary Notes**

**Supplementary Fig. 7** Biexponential fits of the transient PL decay

**Supplementary Fig. 8** Log-log representation of the biexponential fits of the transient PL decay

**Supplementary Table 3** Parameters of the biexponential fits of the transient PL decays

**Supplementary Fig. 9** Transient absorption of HzTFEX_2_ in deaerated and aerated toluene solutions

**Supplementary Fig. 10** Transient absorption decay of HzTFEX_2_ in a deaerated toluene solution at 300 K and 200 K

**Supplementary Fig. 11** Transient PL decays of HzTFEX_2_ in a PPF host matrix at 300 K and 80 K

**Supplementary Fig. 12** Transient EL decays of the OLED using HzTFEX_2_

**Supplementary Table 4** Active-space dependence of the vertical S_1_ and T_1_ excitation energies and Δ*E*_ST_ of HzTFEX_2_ and HzPipX_2_, calculated by CASPT2/cc-pVDZ using CASSCF based on HF orbitals

**Supplementary Table 5** Active-space dependence of the vertical S_1_ and T_1_ excitation energies and Δ*E*_ST_ of HzTFEX_2_ and HzPipX_2_, calculated by CASPT2/cc-pVDZ using CASSCF based on MP2 natural orbitals

**Supplementary Table 6** Vertical S_1_ and T_1_ excitation energies and Δ*E*_ST_ of HzTFEX_2_ and HzPipX_2_, calculated by LT-DF-LCC2/cc-pVDZ and SCS-ADC(2)/cc-pVDZ

**Supplementary Table 7** Vertical S_1_ and T_1_ excitation energies, Δ*E*_ST_, and *f* of HzTFEP_2_ and HzTFET_2_, calculated by various methods

**Supplementary Table 8** Vertical S_1_ and T_1_ excitation energies and Δ*E*_ST_ of heptazine, calculated by various methods with the cc-pVDZ and aug-cc-pVDZ basis sets

**Supplementary Table 9** Compositions of the wave functions in the CASSCF(12,12)/cc-pVDZ calculations

**Supplementary Table 10** Summary of theoretical and experimental excitation energies and Δ*E*_ST_ of HzTFEX_2_, HzPipX_2_, HzTFEP_2_, and HzTFET_2_

**Supplementary Table 11** Vertical S_1_ and T_1_ excitation energies and vertical and adiabatic Δ*E*_ST_ of heptazine, calculated by CASPT2(12,12)/6-31G(d,p)

**Supplementary Table 12** *k*_r_, *k*_nr_, *k*_ISC_ and *k*_RISC_ of HzTFEX_2_ determined by different methods

**1. Supplementary synthesis and characterisation**

**Synthesis of HzPipX_2_** To a solution of 2,5,8-trichloroheptazine (100 mg, 0.362 mmol) in *m*-xylene (3 mL) was added piperidine (36 μL, 0.36 mmol) at room temperature. After 30 min stirring at 100 ºC, the mixture was allowed to cool to room temperature, and AlCl_3_ (106 mg, 0.795 mmol) was added. The reaction mixture was stirred for 1 h at 100 ºC and diluted with H_2_O and CHCl_3_ at room temperature. The organic phase was separated, dried over Na_2_SO_4_, and evaporated. The residue was purified by column chromatography on silica gel (AcOEt/CHCl_3_ = 0:100 to 1:20) to give 19 mg (0.044 mmol, 12%) of yellow solid.

^1^H NMR (600 MHz, CDCl_3_) *δ* [ppm] = 1.68–1.72 (m, 6H), 2.44 (s, 6H), 3.99 (br s, 4H), 7.29 (d, *J* = 7.8 Hz, 4H), 8.44 (d, *J* = 7.8 Hz, 4H). ^13^C NMR (150 MHz, CDCl_3_) *δ* [ppm] = 21.81, 24.32, 26.19, 45.94, 129.16, 130.26, 132.01, 144.76, 156.53, 157.74, 161.11, 174.40. MS (FD-TOF): 464.24440 [M]^+^, calcd. for C_25_H_24_N_8_ (464.24369), error = 1.53 ppm.

**Synthesis of HzTFEX_2_** 60% NaH oil dispersion (121 mg, 3.0 mmol) was added to a solution of 2,2,2-trifluoroethanol (199 μL, 2.78 mmol) in THF (10 mL) at 0 ºC. After stirring for 30 min, the solution was added dropwise to a solution of 2,5,8-trichloroheptazine (700 mg, 2.53 mmol) in THF (20 mL) at 0 ºC. After stirring for 2 h, the mixture was concentrated under reduced pressure to give the yellow solid. The crude products were used in the next step without further purification. The crude products were dissolved in *m*-xylene (12 mL), and AlCl_3_ (1.0 g, 7.5 mmol) was added to the mixture at 0 ºC. The reaction mixture was stirred for 2 h at 0 ºC and then for 17 h at room temperature, and quenched with H_2_O. After an addition of CHCl_3_ and stirring for 30 min, the organic phase was separated, dried over Na_2_SO_4_, and evaporated. The residue was purified by column chromatography on silica gel (CHCl_3_) to give 116 mg (0.242 mmol, 9.6%) of yellow solid.

^1^H NMR (600 MHz, CDCl_3_) *δ* [ppm] = 2.39 (s, 6H), 2.73 (s, 6H), 4.88 (q, *J* = 8.2 Hz, 2H), 7.11–7.13 (m, 4H), 8.19 (d, *J* = 7.8 Hz, 2H). ^13^C NMR (150 MHz, CDCl_3_) *δ* [ppm] = 21.61, 23.15, 126.91, 130.88, 132.49, 133.29, 141.57, 144.46, 157.28, 158.84, 170.12, 178.40. ^19^F NMR (600 MHz, CDCl_3_) *δ* [ppm] = –73.67 (s). MS (FD-TOF): 479.16915 [M]^+^, calcd. for C_24_H_20_F_3_N_7_O (479.16814) , error = 2.1 ppm.

**Synthesis of HzTFET_2_** The synthetic procedure is totally same with that of HzTFEX_2_ except that toluene was used in place of *m*-xylene. ^1^H NMR (600 MHz, CDCl_3_) *δ* [ppm] = 2.46 (s, 6H), 4.90 (q, *J* = 8Hz, 2H), 7.33 (d, *J* = 7.8 Hz, 4H), 8.45 (d, *J* = 8.4 Hz, 4H). ^13^C NMR (150 MHz, CDCl_3_) *δ* [ppm] = 22.20, 129.85, 130.80, 131.12, 146.92, 158.00, 159.17, 169.97, 175.59. ^19^F NMR (600 MHz, CDCl_3_) *δ* [ppm] = –74.12 (s). MS (FD-TOF): 451.13667 [M]^+^, calcd. for C_22_H_16_F_3_N_7_O (451.13684), error = 0.37 ppm.

**Synthesis of HzTFEP_2_** The synthetic procedure is totally same with that of HzTFEX_2_ except that benzene was used in place of *m*-xylene. ^1^H NMR (600 MHz, CDCl_3_) *δ* [ppm] = 4.92 (q, *J* = 8 Hz, 2H), 7.53 (dd, *J* = 7.8 Hz, 4H), 7.68 (dd, *J* = 7.2 Hz, 2H), 8.56 (d, *J* = 7.2 Hz, 4H). ^13^C NMR (150 MHz, CDCl_3_) *δ* [ppm] = 128.78, 130.70, 133.65, 135.07, 158.33, 159.45, 170.22, 176.03. ^19^F NMR (600 MHz, CDCl_3_) *δ* [ppm] = –73.69 (s). MS (FD-TOF): 423.10465 [M]^+^, calcd. for C_20_H_12_F_3_N_7_O (423.10554), error = 2.1 ppm.

**2. Supplementary materials and methods**

**Materials** Commercially available reagents and solvents were used without further purification unless otherwise noted. Poly(3,4-ethylenedioxythiophene):poly(styrene sulfonate) (PEDOT:PSS) aqueous dispersions were purchased from Heraeus. 2,4,5,6-Tetra(carbazol-9-yl)isophthalonitrile (4CzIPN), 4,4′′-bis(triphenylsilyl)-1,1′:4′,1′′-terphenyl (BST), bis(4-(dibenzo[*b*,*d*]furan-4-yl)phenyl)diphenylsilane (DBFSiDBF), bis(diphenylphosphoryl)dibenzo[*b*,*d*]furan (PPF), and 1,3-bis(3,5-di(pyridine-3-yl)phenyl)benzene (B3PyPB) were purchased from Luminescence Technology Corporation. 1,1-Bis(4-di-*p*-tolylaminophenyl)cyclohexane (TAPC), 4,4′-bis(carbazol-9-yl)biphenyl (CBP), tris(8-hydroxyquinolinato)aluminum (Alq3), 8-hydroxyquinolinatolithium (Liq), and 2-methyl-9,10-bis(naphthalene-2-yl)anthracene (MADN) were purchased from e-Ray Optoelectronics Technology. Molybdenum oxide (MoO_3_), lithium fluoride (LiF), and aluminum (Al) were purchased from FURUUCHI Chemical.

**General method** NMR spectra were recorded on a Bruker Avance III HD spectrometer. The chemical shifts (*δ* in ppm) were determined using tetramethylsilane as an internal reference. Matrix-assisted laser desorption ionization time-of-flight (MALDI-TOF) mass spectrometry was performed on a Bruker model AutoflexTM speed spectrometer in the reflector mode using dithranol as a matrix. High-resolution mass data were obtained by using a JEOL JMS-T100GCV with field desorption (FD) as an ionization method.

**OLED fabrication** OLEDs were fabricated on glass substrates covered with indium tin oxide (ITO) (sheet resistance = 15 Ω sq^–1^). The substrates were sequentially cleaned by sonication in detergent, deionized water, acetone, and 2-propanol, followed by UV–ozone treatment for 30 min. PEDOT:PSS was spin-coated on the substrates and annealed at 200 °C on a hot plate for 10 min in ambient conditions. The other materials were sequentially deposited on the substrates under vacuum (< 5 × 10^−5^ Pa) at a deposition rate of < 0.3 nm s^−1^ through shadow masks defining a pixel size of 4.0 mm^2^. The deposition rate and layer thicknesses of each layer was monitored using a quartz crystal microbalance. The devices were ready for evaluation after encapsulation using epoxy glue and glass lids under a nitrogen atmosphere. The fabricated OLEDs consist of the following layer sequences:

Device I. Glass/ITO (130 nm)/PEDOT:PSS (30 nm)/MoO_3_ (5 nm)/BST (3 nm)/DBFSiDBF (10 nm)/PPF:10wt% HzTFEX_2_ (15 nm)/PPF (10 nm)/Alq3 (40 nm)/Liq (1 nm)/Al (80 nm)

Device II. Glass/ITO (130 nm)/PEDOT:PSS (30 nm)/ TAPC (40 nm)/CBP:5wt% 4CzIPN (20 nm)/B3PyPB (40 nm)/LiF (1 nm)/Al (80 nm)

Device III. Glass/ITO (130 nm)/PEDOT:PSS (30 nm)/TAPC (40 nm)/MADN (20 nm)/B3PyPB (40 nm)/LiF (1 nm)/Al (80 nm)

**Supplementary Fig. 1 | Electronic configurations for two electrons in three orbitals.** **a**, Ground state configuration. **b,** Singlet double-excitation configurations. **c**, Triplet double-excitation configuration. The Pauli exclusion principle precludes the two electrons from occupying a given orbital in the triplet double-excitation configuration.

**Supplementary Fig. 2 | Structures of 186 substituents used for generating the candidate heptazine analogues.**

**Supplementary Table 1 |** Vertical S_1_ and T_1_ excitation energies, singlet–triplet energy gap (Δ*E*_ST_), and oscillator strength (*f*) of HzTFEX_2_ and HzPipX_2_, calculated by various methods.

| Molecule | Method | S_1_ excitation  energy (eV) | T_1_ excitation  energy (eV) | Δ*E*_ST_  (meV) | *f* |
| --- | --- | --- | --- | --- | --- |
| HzTFEX_2_ | TDDFT*^a^* | 2.708 | 2.498 | 210 | 0.010 |
|  | EOM-CCSD*^b^* | 2.678 | 2.690 | –12 | 0.019 |
|  | ADC(2)*^c^* | 2.199 | 2.233 | –34 | 0.018 |
|  | CASPT2(12,12)*^d^* | 2.037 | 2.221 | –184 | 0.055 |
| HzPipX_2_ | TDDFT*^a^* | 2.840 | 2.506 | 334 | 0.015 |
|  | EOM-CCSD*^b^* | 3.032 | 3.022 | 10 | 0.040 |
|  | ADC(2)*^c^* | 2.612 | 2.624 | –12 | 0.040 |
|  | CASPT2(12,12)*^d^* | 2.496 | 2.325 | –171 | 0.105 |

*^a^*TDDFT LC-BLYP/6-31G(d) using the geometry optimized for the lowest-energy triplet excited state (T_1_) by unrestricted LC-BLYP/6-31G with the range-separated parameter of 0.18 bour^–1^. *^b^*EOM-CCSD/cc-pVDZ using the T_1_ geometry optimized by unrestricted MP2/cc-pVDZ. *^c^*ADC(2)/cc-pVDZ using the T_1_ geometry optimized by unrestricted MP2/cc-pVDZ. *^d^*CASPT2(12,12)/cc-pVDZ using the T_1_ geometry optimized by unrestricted MP2/cc-pVDZ. The DFT, TDDFT, MP2, and EOM-CCSD calculations were performed using the Gaussian 16 RevC.01 program^1^. The ADC(2) calculations were performed using the Q-Chem 5.3.0 program^2^. The CASSCF and CASPT2 calculations were performed using the Orca 4.2.1 program^3^.

**Supplementary Fig. 3** **| Log-log representation of the transient PL decays of HzTFEX_2_ and HzPipX_2_ at varying temperatures. a**, **b**, Transient PL decays of HzTFEX_2_ (a) and HzPipX_2_ (b) in deaerated toluene at varying temperatures.

**Supplementary Fig. 4 | Rate constants of radiative decay and non-radiative decay (*k*_r_ and *k*_nr_).** Temperature dependence of *k*_r_ + *k*_nr_ of HzTFEX_2_ and HzPipX_2_ determined by numerically fitting Eq. (1) in the main text to the transient PL decays measured in deaerated toluene solutions.

**Supplementary Fig. 5 | Photophysical properties of HzTFEP_2_ and HzPipT_2_.** **a**, Molecular structures of HzTFEP_2_ and HzTFET_2_. **b**, Steady-state absorption and photoluminescence (PL) spectra of HzTFEP_2_ and HzTFET_2_ in deaerated toluene. The inset in (b) is the magnified view of the absorption spectra. **c**, **d**, Transient PL decays of HzTFEP_2_ (c) and HzTFET_2_ (d) at varying temperatures in deaerated toluene. **e**, **f**, Temperature-dependence of the rate constants of ISC and RISC (*k*_ISC_ and *k*_RISC_) of HzTFEP_2_ (e) and HzTFET_2_ (f). The solid lines in (e) and (f) represent the fits of the plots to the Arrhenius equation.

**Supplementary Fig. 6** **| OLED performance. a**, **b**, **c**, **d**, Electroluminescence (EL) spectra measured at 1.0 mA (a), current density–voltage characteristics (b), luminance–voltage characteristics (c), and external quantum efficiency–current density characteristics (d) of the Device I, II, and III.

**Supplementary Table 2** **|** Summary of the OLED performance.

| Device | Emitter | λ_EL_  (nm)*^a^* | CIE  (*x*, *y*)*^b^* | *V*_on_  (V)*^c^* | η_ext,max_  (%)*^d^* | η_ext,1/100/1000_  (%)*^e^* |
| --- | --- | --- | --- | --- | --- | --- |
| I | HzTFEX_2_ | 450, 479 | 0.17, 0.24 | 4.8 | 17.2 | 17.1/10.3/4.2 |
| II | 4CzIPN | 512 | 0.28, 0.56 | 3.2 | 22.5 | 21.4/22.0/19.7 |
| III | MADN | 441 | 0.15, 0.08 | 3.2 | 4.3 | 4.3/3.7/2.0 |

*^a^*Peak wavelength of EL spectra measured at 1.0 mA. *^b^*Commission Internationale de l′éclairage (CIE) coordinates measured at 1.0 mA. *^c^*Turn-on voltage for luminance > 1 cd m^−2^. *^d^*Maximum external quantum efficiency. *^e^*External quantum efficiencies at luminances of 1, 100, and 1000 cd m^−2^.

**3. Supplementary Note**

**Biexponential PL decay analysis.** Biexponential fitting of the transient PL decays was performed using Eq. (S1)


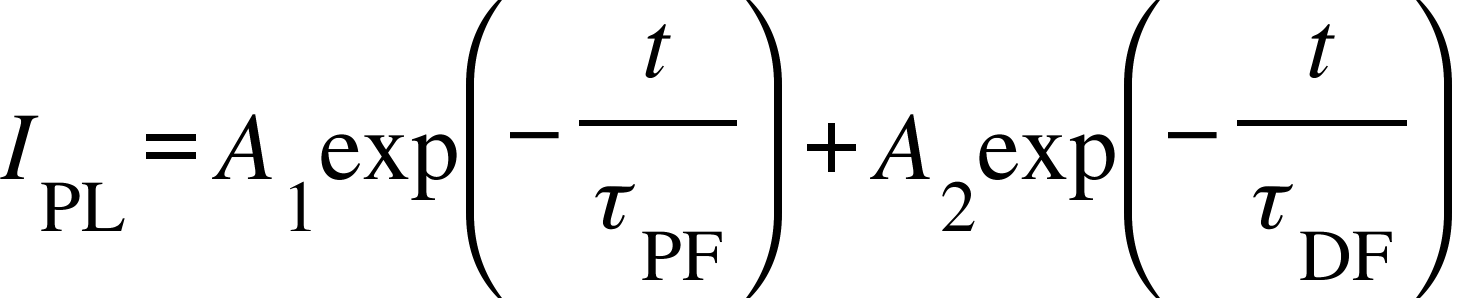
 (S1)

where *I*_PL_ is the PL intensity, τ_PF_ is the time constant of the prompt fluorescence, τ_DF_ is the time constant of the delayed fluorescence, *A* is the pre-exponential factor, and *t* is the time. Using the best-fit parameters in Eq. (S1), the components of prompt fluorescence and delayed fluorescence were simulated using Eq. (S2) and (S3), respectively (Supplementary Figs 7 and 8).


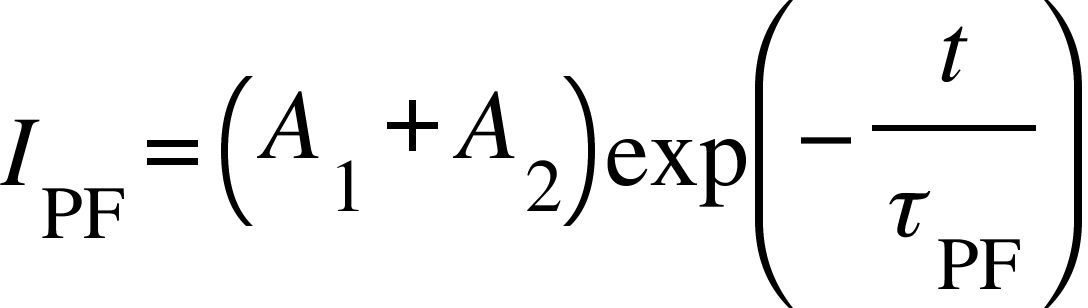
 (S2)

and


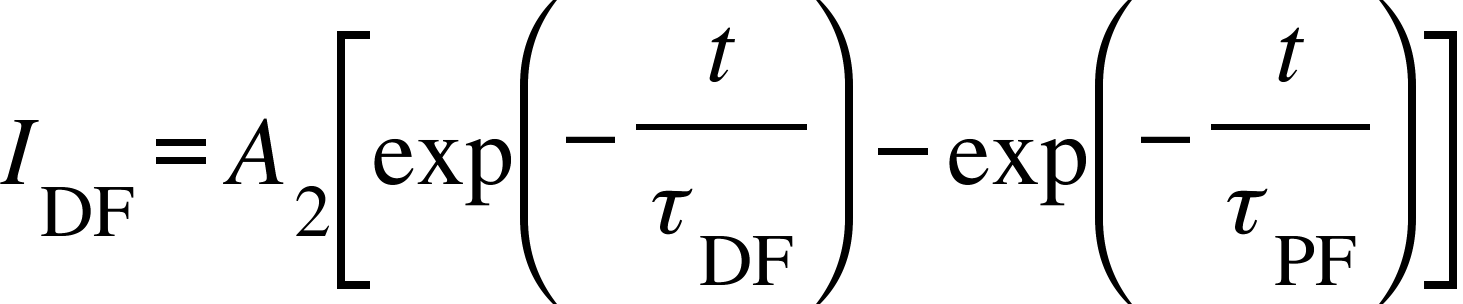
 (S3)

where *I*_PF_ and *I*_DF_ are the intensities of prompt fluorescence and delayed fluorescence, respectively. The obtained fit parameter is listed in Supplementary Table 3.

**Supplementary Fig. 7** **| Biexponential fits of the transient PL decay. a**, **b**, **c**, **d**, Transient PL decays of HzTFEX_2_ (a), HzPipX_2_ (b), HzTFEP_2_ (c), and HzTFET_2_ (d) in deaerated toluene solutions at 300 K. The red solid lines represent biexponential fits of the transient PL decays. The blue and green solid lines represent the components of the prompt fluorescence and the delayed fluorescence, respectively.

**Supplementary Fig. 8** **| Log-log representation of the biexponential fits of the transient PL decay. a**, **b**, **c**, **d**, Transient PL decays of HzTFEX_2_ (a), HzPipX_2_ (b), HzTFEP_2_ (c), and HzTFET_2_ (d) in deaerated toluene solutions at 300 K. The red solid lines represent biexponential fits of the transient PL decays. The blue and green solid lines represent the components of the prompt fluorescence and the delayed fluorescence, respectively.

**Supplementary Table 3 | Parameters of the biexponential fits of the transient PL decays.**

| Emitter | τ_PF_  (ns) | τ_DF_  (ns) | *A*_1_ | *A*_2_ | Φ_PL_  (%)*^a^* | Φ_PF_  (%)*^b^* | Φ_DF_  (%)*^c^* |
| --- | --- | --- | --- | --- | --- | --- | --- |
| HzTFEX_2_ | 14 | 217 | 0.45 | 0.55 | 74 | 8 | 66 |
| HzPipX_2_ | 7.9 | 565 | 0.87 | 0.13 | 67 | 7 | 60 |
| HzTFEP_2_ | 35 | 288 | 0.54 | 0.46 | 44 | 10 | 34 |
| HzTFET_2_ | 23 | 246 | 0.44 | 0.56 | 42 | 7 | 35 |

*^a^*PL quantum yield. *^b^*Component of prompt fluorescence to Φ_PL_. *^c^*Component of prompt fluorescence to Φ_PL_.

**Additional transient absorption measurements.** We performed transient absorption measurements on HzTFEX_2_ in deaerated and aerated toluene solutions (Supplementary Fig. 9). The transient absorption of T_1_ monitored at 1600 nm decayed faster in the aerated solution than the deaerated solution, indicating the quenching the T_1_ by atmospheric O_2_. The quenching of the T_1_ accelerated the S_1_ decay monitored at 700 nm due to the interconversion of S_1_ and T_1_ by ISC and RISC. We also note the S_1_ can be also quenched directly by O_2_^4,5^.

**Supplementary Fig. 9** **| Transient absorption of HzTFEX_2_ in deaerated and aerated toluene solutions.** Transient absorption decays of S_1_ and T_1_ monitored at 700 nm and 1600 nm, respectively, in deaerated and aerated toluene solutions.

Low-temperature transient absorption measurements were also performed on HzTFEX_2_ in a deaerated toluene solution at 200 K (Supplementary Fig. 10). Lowering the temperature from 300 K to 200 K increased the time constant of the initial decay of the transient absorption of S_1_ from 28 ns to 29 ns in the time range of a few tens of nanoseconds. In the sub-microsecond time range, lowering the temperature decreased the time constant of the transient absorption decay of S_1_ from 223 ns to 189 ns. These features are consistent with the trends in the temperature-dependence of the transient PL decay of HzTFEX_2_.

**Supplementary Fig. 10** **| Transient absorption of HzTFEX_2_ in a deaerated toluene solution at 300 K and 200 K.** Transient absorption decays of S_1_ and T_1_ monitored at 700 nm and 1600 nm, respectively, in a deaerated toluene solution at 300 K and 200 K.

**Photophysical properties of HzTFEX_2_ in a solid host matrix.** The photophysical properties of HzTFEX_2_ were evaluated in a PPF host matrix under a N_2_ atmosphere. Upon photoexcitaion a thin film of PPF:1wt% HzTFEX_2_ emits blue emission with two peaks (λ_PL_) at 442 nm and 470 nm, respectively (Extended Data Fig. 4a) and a PL quantum yield (Φ_PL_) of 86%. The transient PL decays comprise nanosecond-order prompt fluorescence followed by sub-microsecond delayed fluorescence (Extended Data Fig. 4b). The time constant of delayed fluorescence (τ_DF_) gradually decreases from 207 ns to 146 ns with lowering the temperature from 300 K to 143 K (Extended Data Fig. 4c). By numerically fitting Eq. (1) in the main text to the transient PL decays at 300 K, *k*_ISC_ and *k*_RISC_ were determined to be 2.6 × 10^7^ s^–1^ and 2.8 × 10^7^ s^–1^. *E*_a,ISC_ and *E*_a,RISC_ were extracted from the temperature dependence of *k*_ISC_ and *k*_RISC_ to be 21 ±1 meV and 12 meV (Extended Data Fig. 4d). Δ*E*_ST_ was determined to be –8 ±1 meV by subtracting *E*_a,ISC_ from *E*_a,RISC_.

The delayed fluorescence of PPF:1wt% HzTFEX_2_ was suppressed at 80 K (Supplementary Fig. 11). This behaviour suggests that ISC is much slower than the radiative decay of S_1_ and the T_1_ population is small at very low temperatures where the thermal energy is too low to overcome *E*_a,ISC_.

**Supplementary Fig. 11** **| Transient PL decays of HzTFEX_2_ in a PPF host matrix at 300 K and 80 K.** Transient PL decays of PPF:1wt% HzTFEX_2_ at 300 K and 80 K under a N_2_ atmosphere.

**Transient electroluminescence (EL) decay at different voltages.** Transient EL decays of the OLED using HzTFEX_2_ were measured in pulse operation with square-wave voltages steps from 5 V to –4 V and from 8 V to –4V, respectively (Supplementary Fig. 12). In both voltage conditions, the device exhibited sub-microsecond transient EL decays, as well as relatively week and long decays in the microsecond time range. Since the negative voltage of –4 V was applied, the recombination of the trapped charges should play a minor role in the long decays. In addition, the long decay component was enhanced at the higher on-voltage (i.e., higher exciton density). It is thus not unreasonable to conclude that the long decay component was caused by bimolecular recombination.

**Supplementary Fig. 12** **| Transient EL decays of the OLED using HzTFEX_2_.** Transient EL decays of the OLED using HzTFEX_2_ measured in pulse operation with square-wave voltages steps from 5 V to –4 V and from 8 V to –4V, respectively.

**Additional CASPT2 calculations.** CASPT2/cc-pVDZ calculations of HzTFEX_2_ and HzPipX_2_ were performed using different active spaces (Supplementary Table 4). The same calculations except that MP2 natural orbitals were used instead of HF orbitals for the state-averaged CASSCF were also performed (Supplementary Table 5). All of the calculations predicted the inversion of S_1_ and T_1_, though the excitation energies and Δ*E*_ST_ are sensitive to the extension of the active space.

**Supplementary Table 4 |** Active-space dependence of the vertical S_1_ and T_1_ excitation energies and Δ*E*_ST_ of HzTFEX_2_ and HzPipX_2_, calculated by CASPT2/cc-pVDZ using CASSCF based on HF orbitals.

| Molecule | Active space | S_1_ excitation  energy (eV) | T_1_ excitation  energy (eV) | Δ*E*_ST_  (meV) |
| --- | --- | --- | --- | --- |
| HzTFEX_2_ | (6,6) | 2.213 | 2.271 | –58 |
|  | (8,8) | 1.719 | 1.759 | –40 |
|  | (10,10) | 1.558 | 1.811 | –253 |
|  | (12,12) | 2.037 | 2.221 | –184 |
| HzPipX_2_ | (6,6) | 1.713 | 3.102 | –1389 |
|  | (8,8) | 2.557 | 2.711 | –154 |
|  | (10,10) | 2.393 | 2.509 | –116 |
|  | (12,12) | 2.496 | 2.325 | –171 |

**Supplementary Table 5 |** Active-space dependence of the vertical S_1_ and T_1_ excitation energies and Δ*E*_ST_ of HzTFEX_2_ and HzPipX_2_, calculated by CASPT2/cc-pVDZ using CASSCF based on MP2 natural orbitals.

| Molecule | Active space | S_1_ excitation  energy (eV) | T_1_ excitation  energy (eV) | Δ*E*_ST_  (meV) |
| --- | --- | --- | --- | --- |
| HzTFEX_2_ | (6,6) | 2.749 | 2.861 | –112 |
|  | (8,8) | 1.592 | 2.736 | –1207 |
|  | (10,10) | 2.283 | 2.371 | –88 |
|  | (12,12) | 1.984 | 2.155 | –171 |
| HzPipX_2_ | (6,6) | 2.822 | 3.048 | –226 |
|  | (8,8) | 2.354 | 2.713 | –359 |
|  | (10,10) | 2.528 | 2.664 | –136 |
|  | (12,12) | 2.423 | 2.701 | –278 |

**Other second-order methods.** Δ*E*_ST_ of HzTFEX_2_ and HzPipX_2_ were also calculated using the Laplace-transformed density-fitted local coupled cluster (LT-DF-LCC2)^6^ and the spin-component-scaled (SCS)-ADC(2)^7^. Both second-order methods predicted the inversion of S_1_ and T_1_ in HzTFEX_2_ and HzPipX_2_ (Supplementary Table 6).

**Supplementary Table 6 |** Vertical S_1_ and T_1_ excitation energies and Δ*E*_ST_ of HzTFEX_2_ and HzPipX_2_, calculated by LT-DF-LCC2/cc-pVDZ and SCS-ADC(2)/cc-pVDZ.

| Molecule | Method | S_1_ excitation  energy (eV) | T_1_ excitation  energy (eV) | Δ*E*_ST_  (meV) |
| --- | --- | --- | --- | --- |
| HzTFEX_2_ | LT-DF-LCC2*^a^* | 2.319 | 2.347 | –28 |
|  | SCS-ADC(2)*^b^* | 2.301 | 2.447 | –146 |
| HzPipX_2_ | LT-DF-LCC2*^a^* | 2.715 | 2.735 | –20 |
|  | SCS-ADC(2)*^b^* | 2.635 | 2.753 | –118 |

*^a^*LT-DF-LCC2/cc-pVDZ using the T_1_ geometry optimized by unrestricted MP2/cc-pVDZ. *^b^*SCS-ADC(2)/cc-pVDZ using the T_1_ geometry optimized by unrestricted MP2/cc-pVDZ. The LT-DF-LCC2 calculations were performed using the MOLPRO 2019.2 program^8^.

**Calculations for HzTFEP_2_ and HzTFET_2_.** Δ*E*_ST_ of HzTFEP_2_ and HzTFET_2_ were calculated using the TDDFT, LT-DF-LCC2, ADC(2), SCS-ADC(2), and CASPT2(12,12). All of the methods except for TDDFT predicted negative Δ*E*_ST_ for both molecules (Supplementary Table 7).

**Supplementary Table 7 |** Vertical S_1_ and T_1_ excitation energies, Δ*E*_ST_, and *f* of HzTFEP_2_ and HzTFET_2_, calculated by various methods.

| Molecule | Method | S_1_ excitation  energy (eV) | T_1_ excitation  energy (eV) | Δ*E*_ST_  (meV) | *f* |
| --- | --- | --- | --- | --- | --- |
| HzTFEP_2_ | TDDFT*^a^* | 2.729 | 2.464 | 265 | 0.008 |
|  | EOM-CCSD*^b^* | 2.892 | 2.925 | –33 | 0.013 |
|  | LT-DF-LCC2*^c^* | 2.650 | 2.765 | –115 | – |
|  | ADC(2)*^d^* | 2.156 | 2.197 | –41 | 0.019 |
|  | SCS-ADC(2)*^e^* | 2.545 | 2.878 | –333 | 0.015 |
|  | CASPT2(12,12)*^f^* | 2.108 | 2.463 | –355 | 0.167 |
| HzTFET_2_ | TDDFT*^a^* | 2.748 | 2.489 | 259 | 0.009 |
|  | EOM-CCSD*^b^* | 2.899 | 2.944 | –45 | 0.011 |
|  | LT-DF-LCC2*^c^* | 2.696 | 2.786 | –90 | – |
|  | ADC(2)*^d^* | 2.147 | 2.229 | –82 | 0.025 |
|  | SCS-ADC(2)*^e^* | 2.601 | 2.867 | –266 | 0.014 |
|  | CASPT2(12,12)*^f^* | 2.192 | 2.456 | –264 | 0.198 |

*^a^*TDDFT LC-BLYP/6-31G(d) using the geometry optimized for T_1_ by unrestricted LC-BLYP/6-31G with the range-separated parameter of 0.18 bour^–1^. *^b^*EOM-CCSD/cc-pVDZ using the T_1_ geometry optimized by unrestricted MP2/cc-pVDZ. *^c^*LT-DF-LCC2/cc-pVDZ using the T_1_ geometry optimized by unrestricted MP2/cc-pVDZ. *^d^*ADC(2)/cc-pVDZ using the T_1_ geometry optimized by unrestricted MP2/cc-pVDZ. *^e^*SCS-ADC(2)/cc-pVDZ using the T_1_ geometry optimized by unrestricted MP2/cc-pVDZ. *^f^*CASPT2(12,12)/cc-pVDZ using the T_1_ geometry optimized by unrestricted MP2/cc-pVDZ.

**Diffuse basis function.** Δ*E*_ST_ of heptazine were calculated using the EOM-CCSD, LT-DF-LCC2, ADC(2), SCS-ADC(2), and CASPT2(12,12) with the diffuse-function-augmented aug-cc-pVDZ basis set. For each method, the Δ*E*_ST_ values appear not to be sensitive to the extension of the basis set from cc-pVDZ to aug-cc-pVDZ (Supplementary Table 8).

**Supplementary Table 8 |** Vertical S_1_ and T_1_ excitation energies and Δ*E*_ST_ of heptazine, calculated by various methods with the cc-pVDZ and aug-cc-pVDZ basis sets.

| Method*^a^* | Basis | S_1_ excitation  energy (eV) | T_1_ excitation  energy (eV) | Δ*E*_ST_  (meV) |
| --- | --- | --- | --- | --- |
| EOM-CCSD | cc-pVDZ | 2.656 | 2.838 | –182 |
|  | aug-cc-pVDZ | 2.727 | 2.884 | –157 |
| LT-DF-LCC2 | cc-pVDZ | 2.552 | 2.817 | –265 |
|  | aug-cc-pVDZ | 2.572 | 2.831 | –259 |
| ADC(2) | cc-pVDZ | 2.438 | 2.723 | –285 |
|  | aug-cc-pVDZ | 2.474 | 2.743 | –269 |
| SCS-ADC(2) | cc-pVDZ | 2.364 | 2.895 | –531 |
|  | aug-cc-pVDZ | 2.428 | 2.926 | –498 |
| CASPT2(12,12) | cc-pVDZ | 2.302 | 2.511 | –209 |
|  | aug-cc-pVDZ | 2.259 | 2.395 | –136 |

*^a^*All of the calculations used the T_1_ geometry optimized by unrestricted MP2/cc-pVDZ.

**Composition of CASSCF wave function.** The compositions of the wave functions in the CASSCF(12,12)/cc-pVDZ calculations of HzTFEX_2_, HzPipX_2_, HzTFEP_2_, and HzTFET_2_ are listed in Supplementary Table 9. The S_0_ of the four molecules are almost described by the closed-shell configuration. The S_1_ of the four molecules are dominated by the single-excitation configuration with the small contributions from the multiple-excitation configuration, which are slightly higher than those of T_1_.

**Supplementary Table 9 |** Compositions of the wave functions in the CASSCF(12,12)/cc-pVDZ calculations.

| Molecule | State | Configuration | Weight (%)*^a^* |
| --- | --- | --- | --- |
| HzTFEX_2_ | S_0_ | Closed shell | 86.9 |
|  |  | Single excitation | 4.1 |
|  |  | Multiple excitation | 5.3 |
|  | S_1_ | Closed shell | 3.0 |
|  |  | Single excitation | 86.0 |
|  |  | Multiple excitation | 8.5 |
|  | T_1_ | Closed shell | – |
|  |  | Single excitation | 89.7 |
|  |  | Multiple excitation | 7.5 |
| HzPipX_2_ | S_0_ | Closed shell | 83.4 |
|  |  | Single excitation | 3.4 |
|  |  | Multiple excitation | 5.0 |
|  | S_1_ | Closed shell | 2.8 |
|  |  | Single excitation | 79.1 |
|  |  | Multiple excitation | 8.9 |
|  | T_1_ | Closed shell | – |
|  |  | Single excitation | 83.9 |
|  |  | Multiple excitation | 8.0 |
| HzTFEP_2_ | S_0_ | Closed shell | 81.8 |
|  |  | Single excitation | 3.7 |
|  |  | Multiple excitation | 2.8 |
|  | S_1_ | Closed shell | 1.4 |
|  |  | Single excitation | 77.5 |
|  |  | Multiple excitation | 8.8 |
|  | T_1_ | Closed shell | – |
|  |  | Single excitation | 83.2 |
|  |  | Multiple excitation | 6.3 |
| HzTFET_2_ | S_0_ | Closed shell | 81.8 |
|  |  | Single excitation | 3.9 |
|  |  | Multiple excitation | 2.5 |
|  | S_1_ | Closed shell | 1.5 |
|  |  | Single excitation | 77.2 |
|  |  | Multiple excitation | 7.6 |
|  | T_1_ | Closed shell | – |
|  |  | Single excitation | 83.2 |
|  |  | Multiple excitation | 4.9 |

*^a^*Sum of the configuration weights of > 0.25%.

**Supplementary Table 10 |** Summary of theoretical and experimental excitation energies and Δ*E*_ST_ of HzTFEX_2_, HzPipX_2_, HzTFEP_2_, and HzTFET_2_.

| Molecule | Method | S_1_ excitation  energy (eV) | T_1_ excitation  energy (eV) | Δ*E*_ST_  (meV) |
| --- | --- | --- | --- | --- |
| HzTFEX_2_ | TDDFT | 2.708 | 2.498 | 210 |
|  | EOM-CCSD | 2.678 | 2.690 | –12 |
|  | LT-DF-LCC2 | 2.319 | 2.347 | –28 |
|  | ADC(2) | 2.199 | 2.233 | –34 |
|  | SCS-ADC(2) | 2.301 | 2.447 | –146 |
|  | CASPT2(12,12) | 2.037 | 2.221 | –184 |
|  | Experiment*^a^* | 2.76 | 2.77 | –11 ± 2 |
| HzPipX_2_ | TDDFT | 2.840 | 2.506 | 334 |
|  | EOM-CCSD | 3.032 | 3.022 | 10 |
|  | LT-DF-LCC2 | 2.715 | 2.735 | –20 |
|  | ADC(2) | 2.612 | 2.624 | –12 |
|  | SCS-ADC(2) | 2.635 | 2.753 | –118 |
|  | CASPT2(12,12) | 2.496 | 2.325 | –171 |
|  | Experiment*^a^* | 2.81 | 2.76 | 52 ± 1 |
| HzTFEP_2_ | TDDFT | 2.729 | 2.464 | 265 |
|  | EOM-CCSD | 2.892 | 2.925 | –33 |
|  | LT-DF-LCC2 | 2.650 | 2.765 | –115 |
|  | ADC(2) | 2.156 | 2.197 | –41 |
|  | SCS-ADC(2) | 2.545 | 2.878 | –333 |
|  | CASPT2(12,12) | 2.108 | 2.463 | –355 |
|  | Experiment*^a^* | 2.73 | 2.72 | –14 ± 3 |
| HzTFET_2_ | TDDFT | 2.748 | 2.489 | 259 |
|  | EOM-CCSD | 2.899 | 2.944 | –45 |
|  | LT-DF-LCC2 | 2.696 | 2.786 | –90 |
|  | ADC(2) | 2.147 | 2.229 | –82 |
|  | SCS-ADC(2) | 2.601 | 2.867 | –266 |
|  | CASPT2(12,12) | 2.192 | 2.456 | –264 |
|  | Experiment*^a^* | 2.75 | 2.74 | –13 ± 3 |

*^a^*Experimental S_1_ excitation energy was estimated from the lowest-energy peak of the fluorescence spectra. Δ*E*_ST_ was obtained as the difference between the activation energies of ISC and RISC. The T_1_ excitation energy was estimated by subtracting Δ*E*_ST_ from the S_1_ excitation energy.

**Adiabatic Δ*E*_ST_ of heptazine.** The S_1_ and T_1_ geometries of heptazine were optimised by CASSCF(12,12)/6-31G(d,p). The vertical excitation energies and Δ*E*_ST_ were calculated by CASPT2(12,12)/6-31G(d,p) with a more contracted configuration space^9^. Adiabatic Δ*E*_ST_ was obtained as the difference between the total CASPT2 energies of the S_1_ and T_1_ at their optimised geometries. The CASPT2 calculations predicted negative Δ*E*_ST_ at both S_1_ and T_1_ geometries, as well as negative adiabatic Δ*E*_ST_ (Supplementary Table 11).

**Supplementary Table 11** | Vertical S_1_ and T_1_ excitation energies and vertical and adiabatic Δ*E*_ST_ of heptazine, calculated by CASPT2(12,12)/6-31G(d,p).

| Geometry*^a^* | S_1_ excitation  energy (eV) | T_1_ excitation  energy (eV) | Vertical Δ*E*_ST_  (meV) | Adiabatic Δ*E*_ST_  (meV) |
| --- | --- | --- | --- | --- |
| S_1_ geometry | 2.305 | 2.517 | –212 | –371 |
| T_1_ geometry | 2.185 | 2.350 | –165 |  |

*^a^*Optimised by the state-averaged (S_0_, S_1_ and T_1_) CASSCF(12,12)/6-31G(d,p) calculations implemented in the MOLPRO 2019.2 program^8^.

**Confirming the rate inversion of ISC and RISC with an alternative method.** The excited-state kinetics of HzTFEX_2_ in deaerated toluene solutions at 300 K was also analysed by the method reported by Tsuchiya *et al*.^10^ As consistent with the results discussed in the main text, Tsuchiya’s method also revealed the rate inversion of ISC and RISC (i.e. *k*_RISC_ > *k*_ISC_).

**Supplementary Table 12** | *k*_r_, *k*_nr_, *k*_ISC_ and *k*_RISC_ of HzTFEX_2_ determined by different methods.

| Method | *k*_r_ (s^–1^) | *k*_nr_ (s^–1^) | *k*_ISC_ (s^–1^) | *k*_RISC_ (s^–1^) |
| --- | --- | --- | --- | --- |
| Fit using Eq. (1) | 5.4 × 10^6^ | 1.9 × 10^6^ | 2.3 × 10^7^ | 4.2 × 10^7^ |
| Tsuchiya’s method | 5.9 × 10^6^ | 2.0 × 10^6^ | 2.6 × 10^7^ | 4.1 × 10^7^ |

**4. References**

1 Gaussian 16, Revision C.01, Frisch, M. J. et al. Gaussian, Inc., Wallingford CT, 2016.

2 Shao, Y. et al. Advances in molecular quantum chemistry contained in the Q-Chem 4 program package. *Mol. Phys.* **113**, 184–215 (2015).

3 Neese, F. Software update: the ORCA program system, version 4.0, *Wiley Interdiscip. Rev.: Comput. Mol. Sci.*, **8**, e1327 (2017).

4 Gollnick, K. & Schenck, G.O. Mechanism and stereoselectivity of photosensitized oxygen transfer reactions, *Pure Appl. Chem.* **9**, 507–526 (1964).

5 Kikuchi, K., Sato, C., Watabe, M., Ikeda, H., Takahashi, Y. & Miyashi, T. New Aspects on Fluorescence Quenching by Molecular Oxygen, *J. Am. Chem. Soc*. **115**, 5180–5184 (1993).

6 Kats, D. & Schütz, M. A multistate local coupled cluster CC2 response method based on the Laplace transform, *J. Chem. Phys.* **131**, 124117 (2009).

7 Krauter, C. M., Pernpointner, M. & Dreuw, A. Application of the scaled-opposite-spin approximation to algebraic diagrammatic construction schemes of second order, *J. Chem. Phys.* **138**, 044107 (2013).

8 Werner, H. et al. The Molpro quantum chemistry package, *J. Chem. Phys.* **152**, 144107 (2020).

9 Celani, P. & Werner H.-J. Multireference perturbation theory for large restricted and selected active space reference wave functions, *J. Chem. Phys.* **112**, 5546 (2000).

10 Tsuchiya, Y. *et al.* Exact Solution of Kinetic Analysis for Thermally Activated Delayed Fluorescence Materials, *J. Phys. Chem. A* **125**, 8074–8089 (2021).
